# Molecular Characterization of the *Aphis gossypii* Olfactory Receptor Gene Families

**DOI:** 10.1371/journal.pone.0101187

**Published:** 2014-06-27

**Authors:** Depan Cao, Yang Liu, William B. Walker, Jianhong Li, Guirong Wang

**Affiliations:** 1 Laboratory of Pesticide, College of Plant Science & Technology, Huazhong Agricultural University, Wuhan, China; 2 State Key Laboratory for Biology of Plant Diseases and Insect Pests, Institute of Plant Protection, Chinese Academy of Agricultural Sciences, Beijing, China; 3 Swedish University of Agricultural Sciences, Department of Plant Protection Biology, Chemical Ecology Research Group, Alnarp, Sweden; United States Department of Agriculture, Beltsville Agricultural Research Center, United States of America

## Abstract

The cotton aphid, *Aphis gossypii* Glover, is a polyphagous pest that inflicts great damage to cotton yields worldwide. Antennal olfaction, which is extremely important for insect survival, mediates key behaviors such as host preference, mate choice, and oviposition site selection. In insects, odor detection is mediated by odorant receptors (ORs) and ionotropic receptors (IRs), which ensure the specificity of the olfactory sensory neuron responses. In this study, our aim is to identify chemosensory receptors in the cotton aphid genome, as a means to uncover olfactory encoding of the polyphagous feeding habits as well as to aid the discovery of new targets for behavioral interference. We identified a total of 45 candidate *ORs* and 14 *IRs* in the cotton aphid genome. Among the candidate *AgoORs*, 9 are apparent pseudogenes, while 19 can be clustered with *ORs* from the pea aphid, forming 16 *AgoOR/ApOR* orthologous subgroups. Among the candidate *IRs*, we identified homologs of the two highly conserved co-receptors *IR8a* and *IR25a*; no AgoIR retain the complete glutamic acid binding domain, suggesting that putative AgoIRs bind different ligands. Our results provide the necessary information for functional characterization of the chemosensory receptors of *A. gossypii*, with potential for new or refined applications of semiochemicals-based control of this pest insect.

## Introduction

Chemical senses are critical for most animals, due to the fact that most key behaviors, such as foraging, mating and predator-avoidance, are strongly dependant upon chemical sensing. The importance is most significantly realized in insects by their iconic protuberant antenna, which is the primary olfactory organ. The chemosensory structure, sensilla, can be found across the whole body of the insect. Most sensilla are distributed on the surface of olfactory organs, such as antennae, mouth parts, legs, wings and ovipositor [1]. Olfactory receptor neurons (ORNs) are the terminal interfaces of the chemical sensing systems, which are hosetd in the root of sensilla and project their dendrites into the cavity of sensilla. The chemosensory receptors (CR), which are located within the cell membrane of the ORN dendrite, confer the specificity of the OSN olfactory responses.

The insect chemoreceptor superfamily was first identified in the *Drosophila melanogaster* genome and consists of the odorant receptor (*OR*) family and the gustatory receptor (*GR*) family. Genes from these families are expressed at low levels in the antennae, maxillary palps, and other tissues [2]. These seven-transmembrane-domain (7TM) proteins [3]–[6] were thought to be ligand-gated ion channels, which are evolutionarily distant from the vertebrate G-protein–coupled receptors (GPCRs) chemosensory receptor family [7]–[13]. In 2009, a different class of ligand-gated ion channel, the ionotropic receptor (*IR*), was identified as an additional repertoire of chemoreceptors of *ORs* and *GRs* [14]. The *IRs* belong to a variant subfamily of ionotropic glutamate receptors (*iGluRs*), which is best known for their role in allowing neurons to communicate with each other in the brain. Evidence of highly divergent *iGluRs*, expressing in olfactory organs, with chemosensory sensillla subcellular localization combined with mis-expression experiments has provided direct genetic evidence for the role of *IRs* in odor sensing [15].

In *D. melanogaster*, 62 olfactory receptors (*OR*) and 68 gustatory receptors (*GR*) were detected, with alternative splice variants also identified. Comparison to the 79 *ORs* and 76 *GRs* from *Anopheles gambiae* revealed that *GRs* of the heteromeric carbon dioxide receptors and several sugar receptors are conserved [16]–[18] but *ORs* of different species are highly divergent, and that few simple orthologous relationships remained. One exception is the *DmOR83b* and its ortholog in *A. gambiae AgOR7*, which function as a heterodimerization partner for all of the other ORs and is broadly conserved [19], [20]. This species-specific expansion trend of *ORs* is confirmed by genome sequencing of other insects. Within the genomes of *Bombyx mori, Apis mellifera, Aedes aegypti,* and *Nasonia vitripennis*, 41, 170, 131 and 301 *ORs* were respectively identified [21]–[24]. All of these works demonstrate that the *OR* family undergoes rapid evolution in a species-specific manner, with the exception of the *DmOR83b* orthologs, now referred to as *Orco*, to reflect their funciton as the OR co-receptor. Recent transcriptome works on Lepidoptera sexual pheromone receptors, however, provide an example of inter-species *OR* homologs. As that Lepidopterans use long chain polyunsaturated fat acid analogues for their sexual pheromone communication, the Lepidopterans’ pheromone receptor genes are conserved enough that they can be grouped together as a subfamily of *ORs* in inter-species phylogenetic analyses [25]. Aphids are model organisms in both evolutionary and applied biology. For evolutionary biologists, the extended group of more than 4,000 species and specialization to their host plants make aphids the perfect model to study evolution and coevolution between plants and herbivorous insects [26], [27]. For example, genetic differences have been reported between *Acyrthosiphon pisum* host plant races with reproductive isolation occurring as by-product of host adaptation [28], [29]. For applied biology, aphids represent one of the major pests in agriculture, with the characteristics of rapid breeding, causing great economic losses and serious insecticide dependence for population control.

The cotton aphid, *Aphis gossypii* Glover, is a polyphagous pest that damages cotton through direct feeding on the underside of leaves or on the growing tips of shoots. Feeding is carried out through the sucking of juices from the plant, causing leaf curling and distortion, which greatly hinders efficient photosynthesis, and induces foliage chlorotic and premature death. Unlike the monophagous Pea aphid *(Acyrthosiphon pisum)*, the cotton aphid has a very wide host range of over 700 species world-wide, including watermelons, cucumbers, pumpkin, pepper, eggplant, okra and hibiscus [30]. As the genome of the cotton aphid had been sequenced and is under continuous refinement, we report our chemosensory receptor gene analysis of 45 *ORs* and 13 *IRs*, and the expression profiles of these families.

## Materials and Methods

### Identification of cotton aphid *ORs* and *IRs* by bioinformatics

The 3^rd^ assembly version of *A. gossypii* genome was queried using previously described *OR* sequences from *A.pisum* [31], *An. Gambiae* [32], *D. melanogaster* [4], [33], [34] and other known *ORs* and *IRs* from GenBank by tblastn, in order to obtain possible *OR* and *IR* exons. Genomic scaffold sequences of exons found in tblastn were used to construct putative *OR* and *IR* sequences manually using Sequencher v4.5 (Gene Codes, Inc., Ann Arbor, MI) and refined using SplicePredictor (http://deepc2.psi.iastate.edu/cgi-bin/sp.cgi/). All *A. gossypii ORs* identified in this manner were in turn used in successive tblastn searches to identify other candidate sequences, which were annotated as above. Cotton aphid OR genes were named as “AgoOR” with a number which is confirmed by similarity with Pea aphid *ORs*. Due to high divergence and/or discontinuity among scaffolds, not all detected *OR/IR* genes could be entirely annotated: In these cases, deduced amino acid sequences shorter than 150 amino acids were discarded as probable gene fragments, while for sufficiently long partial sequences a suffix N, C or M after the gene–protein name was used to indicate that the 5′ terminus (N) or 3′ terminus (C) or the internal exon (M) is missing. When frameshifts or stop codons were identified in the gene sequence, we defined these genes as putative pseudogenes (suffix P). As highly similar gene models were found in our annotations, pairwise alignment was used to detect single nucleotide differences and non-frameshift gaps. If one sequence had only non-frameshift gaps and located on a separate small contig, they were considered to be likely allelic variants and excluded from the final list. If some sequences were located in long tandem arrays in one contig, they were considered to be paralogs and were kept, ignoring their similarities to other sequences. For highly divergent sequence detection, artificial gene models were used as queries in PSI-blastp searches against the *A. gossypii* unigenes database and genome annotation database. Sequences detected in PSI-blastp were mapped to the genome to confirm and extend the artificial annotation. The final annotations of artificial gene models were supported to be *OR/IR* by matches to other insect CRs using blastp against the NCBI non-redundant protein database (nr). *AgoORs* were confirmed by their trans-member structures using TMHMM [6].

### Intron/exon analysis

The predicted structure of each *AgoOR* gene was reconstructed and recorded as described above. The structure of each Pea Aphid odorant receptors were obtained by mapping *ApOR* sequences to the Pea aphid genome assembly version 2 (http://www.aphidbase.com/aphidbase/downloads) with GMAP [35]. The splice sites of *AgoORs* and *ApORs* nucleotide sequences were transformed into deduced amino acid coordinates with ORF phase indicated. Deduced amino acid sequences of *AgoORs* were aligned with *ApORs* using ClustalW [36]. Then the splice positions of *AgoORs* and *ApORs* were transformed into amino acid coordinates and mapped to the aligned peptide sequences. Only the most parsimonious intron locations were considered to be conserved. All other were considered idiosyncratic.

### Phylogenetic analysis

Deduced amino acid sequences of *AgoORs* were aligned with *ApORs* by ClustalW using default settings. Corrected distances were obtained using the maximum likelihood method in MEGA5.10 [37] with the Jones-Taylor-Thornton amino acid substitution model (JTT model) and otherwise default settings. Node support was assessed using a bootstrap procedure based on 1000 replicates. The *AgoIR* phylogenetic analysis was performed as above, while the reference dataset contained 12, 18, and 66 *IR* sequences from *S. littoralis*, *B. mori, D. melanogaster*, respectively and 10 *iGluRs* sequences from *D. melanogaster* [25].

### Quantitative real-time PCR (qRT-PCR) analysis

Cotton aphids from a laboratory colony, which has been raised on cotton seedling for over ten years, were used in our qRT-PCR analysis. Different parts and organs of cotton aphid were collected. The forepart of the aphid head was collected by clipping beneath the ommateum using a precise scissors. This part containing antenna, proboscis and part of the aphid head, was marked as “head”. The aphid “leg” sample was collected by directly tweezing from the very root of the legs. The aphid “body” sample contains the thorax and abdomen without legs.

Total RNA of different samples was extracted separately using TRIZOL, and 2 µg total RNA of each sample was reverse-transcribed by the One-Step gDNA Removal and cDNA Synthesis kit (TRANSGEN, China) using Ploy-T primer. qRT-PCR was performed on ABI Prism 7500 real-time PCR system (Applied Biosystems, Foster City, CA, USA) using the GoTaq qPCR Master Mix (Promega), according to the manufacturers’ instructions. The first-strand cDNA (2 µl) and the no-template control (NTC, 2 µl) were used as templates for three technical replication assays in each 20 µl reaction mixture under the following conditions: denaturation at 95°C for 60 s, followed by 40 cycles of 95°C for 15 s and 60°C for 60 s. After amplification, melting curves were constructed of the temperature range 60–95°C and data analysis was performed on SDS software with the ABI 7500 system. The results were standardized to the expression level of cotton aphid GAPDH gene. The 2^−ΔΔCt^ method [38] was used to analyze the relative differences in the transcript levels.

## Results

### The odorant receptor family

We identified a total of 45 candidate *AgoOR* genes in the genome of *A. gossypii*. Of these, 22 genes encode putative, complete functional proteins, 14 are incompletely annotated genes, and 9 are apparent pseudogenes (Table 1). As is well-known, a reliable homology based gene prediction is heavily dependent on the maturity of the genome assembly. That means the inevitable refinement of genome assembly will ultimately result in changes in the known complexity of the *AgoOR* gene family. But according to the current available assembly version 3, these represent the complete *OR* repertoire in the *A. gossypii* genome. During our gene prediction procedure, more than 100 sites in the genome displayed potential homology with queried OR sequences. However, half of these sites were located on small scaffolds with length less than 10K nt, which produced incomplete ORFs or short coding fragments. Most of these fragments were filtered as redundant sequences because of their high similarity with other *AgoORs*. The sequences of putative *AgoOR*s are attached in Supplementary Material S1.

**Table 1:** Summary of putative odorant receptor genes of A. *gossypii.*

Gene	Length	Status	Most similar ApOR	%identity	tm domains
AgoOrco	1383	Complete ORF	ApOR1	0.95	7
AgoOR2	1212	5′ exon lost	ApOR2	0.843	6
AgoOR3	1146	Complete ORF	ApOR4	0.422	6
AgoOR4	1164	Complete ORF	ApOR4	0.801	7
AgoOR5	723	Internal exon lost	ApOR5	0.505	6
AgoOR6	1308	Complete ORF	ApOR64	0.594	6
AgoOR7	1242	Complete ORF	ApOR32	0.498	9
AgoOR8	1108	Pseudogene	ApOR32	0.444	6
AgoOR9	948	Internal exon lost	ApOR9	0.53	3
AgoOR10	1119	Complete ORF	ApOR10	0.721	6
AgoOR11	933	5′ exon lost	ApOR23	0.322	4
AgoOR12	1239	Complete ORF	ApOR43	0.561	6
AgoOR13	1068	Pseudogene	ApOR43	0.68	8
AgoOR14	795	5′,3′ exon lost	ApOR32	0.316	4
AgoOR15	1077	Complete ORF	ApOR23	0.373	6
AgoOR16	807	Pseudogene	ApOR32	0.316	4
AgoOR17	984	Internal exon lost	ApOR17	0.366	4
AgoOR18	450	5′ exon lost	ApOR17	0.206	2
AgoOR19	654	5′,3′ exon lost	ApOR22	0.44	3
AgoOR20	1383	Complete ORF	ApOR20	0.76	6
AgoOR21	1088	Pseudogene	ApOR21	0.67	5
AgoOR22	534	5′,3′ exon lost	ApOR22	0.343	4
AgoOR23	1293	Complete ORF	ApOR23	0.753	7
AgoOR24	1122	Complete ORF	ApOR24	0.573	5
AgoOR25	1068	Complete ORF	ApOR25	0.577	6
AgoOR26	1386	Complete ORF	ApOR25	0.566	8
AgoOR27	945	5′ exon lost	ApOR23	0.333	3
AgoOR28	720	Internal exon lost	ApOR32	0.301	4
AgoOR29	1338	Complete ORF	ApOR25	0.581	6
AgoOR30	1335	Pseudogene	ApOR25	0.661	6
AgoOR31	1219	Pseudogene	ApOR31	0.686	6
AgoOR32	801	Internal exon lost	ApOR31	0.448	5
AgoOR33	849	Internal exon lost	ApOR33	0.34	4
AgoOR34	909	3′ exon lost	ApOR32	0.333	7
AgoOR35	1167	Complete ORF	ApOR35	0.563	6
AgoOR36	1174	Pseudogene	ApOR35	0.402	6
AgoOR37	1155	Complete ORF	ApOR37	0.796	6
AgoOR38	1230	5′ lost	ApOR38	0.695	7
AgoOR39	1146	Complete ORF	ApOR39	0.776	4
AgoOR40	540	5′ exon lost	ApOR40	0.319	2
AgoOR41	1260	Complete ORF	ApOR38	0.724	7
AgoOR42	1288	Pseudogene	ApOR42	0.721	7
AgoOR43	1257	Complete ORF	ApOR43	0.806	7
AgoOR44	1288	Pseudogene	ApOR45	0.543	6
AgoOR45	1119	Complete ORF	ApOR45	0.485	6

Due to the high divergence of insect *ORs* as well as genome complexites, full-length annotation of *AgoORs* is difficult. In our study, 7 gene models are incompletely annotated as a result of failure to detect 5′ and 3′ ends. *AgoOR18, 22* and *40* are short gene models consisting of only one or two recognizable exons and encode peptides less than 200aa; *AgoOR11* and *34* are relatively long gene models, located on big scaffolds and near other *AgoORs*, but their N and C terminal ends remain undetected. Large introns, assembly errors and sequencing gaps may also result in incomplete gene models. *AgoOR5, 9, 17, 19, 28, 32* and *33* are marked incomplete as there are unknown nucleotides (Ns) in their exons or are apparently matched to the terminal ends of known insect *OR* but the internal sequence remains unidentified. These gene models also failed in the TMHMM test, as intact *OR* genes should have a hallmark of seven-transmembrane-domains (Table 1).

Pseudogenes are another reason for incomplete sequences. Notably, we identified 9 potential *pseudo-ORs* out of 45 odorant receptors, which is reflective of a high rate of pseudoenization. All pseudogenes are the result of frameshifts caused by indels in their open-reading frames. Pseudogenes are rare occurrences in model insects like *D. melanogaster* and *An. gambiae* genomes [39]. In pea aphid genome there are also 10 *pseudo-ORs* detected, but the total number of *ApORs* is 73, nearly twice that of the *OR* family of cotton aphid. This phenomenon is likely to be caused by sequencing errors or misassemblies in the cotton aphid genome.

Amino acid identity percentage between genes represents the similarity of two genes. We collected the identity data of *AgoORs* versus *ApORs*. As a result, the average identity percentage of all 45 *AgoORs* versus *ApORs* is 54.76%; the average identity of intact genes is 64.08%. 26 *AgoORs* have identities higher than 50%. By phylogenetic analysis of the proteins encoded by the 45 *AgoORs* along with the 73 *ApORs*, we identified 16 *AgoOR/ApOR* orthologous subgroups, which contain 19 *AgoOR* in total. Orthologous subgroup with the highest identity, 95.0%, is the *AgoOrco/ApOR1* subgroup, representing the highly conserved Orco genes including the Drosophila Orco (*OR83b*) gene, *Ae. aegypti* and *An. gambiae* Orco (*OR7*) genes and *B. mori* Orco (*OR2*) gene. These two genes are clear orthologs as prior research in *Drosophila* have proven that Orco are essential for olfactory signal transduction [8], [19]. Other than the *Orco* family, orthologous subgroups with identities higher that 80% include the *AgoOR2/ApOR2*, *AgoOR43/ApOR43* and *AgoOR4/ApOR4* subgroups with 84.3%, 80.6%, and 80.1% identites respectively. Another eight *AgoOR* sequences (*AgoOR 10, 20, 23, 37, 38, 39, 41* and *42*) have closely related *ApOR* homologues, with identities greater than 70%.

**Figure 1:**
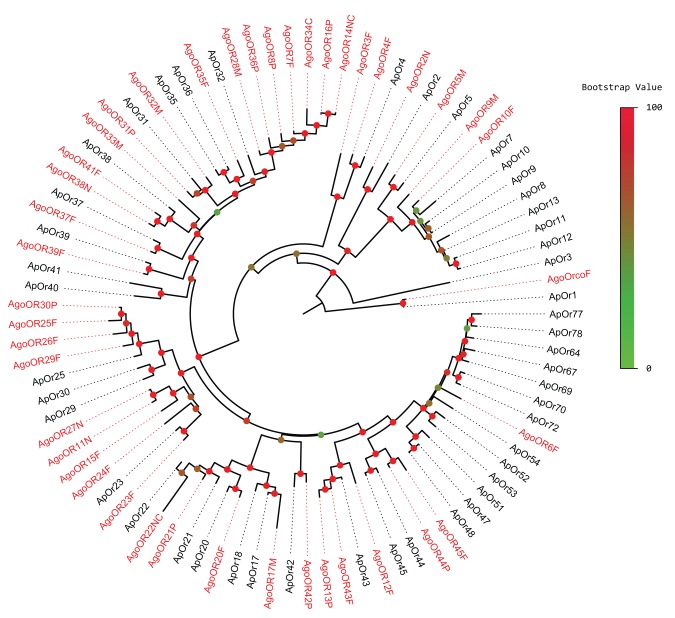
Phylogenetic relationships of the cotton aphid *AgoORs* with relative pea aphid *ApORs*. This corrected distance tree was rooted by declaring the *Orco* gene orthologs (*ApOR1–AgoOrco* clade) as outgroup, based on the basal location of the *Orco* within the *OR* family. Support for major branches is shown above them as percent of 1,000 uncorrected distance bootstrap replications. Suffixes after gene names are: P–pseudogene, N–N-terminal exon(s) or region missing, C–C-terminal exon(s) or region missing, M–internal exon(s) or region missing. Species abbreviations are Ap – *Acyrthosiphon pisum* and Ago – *Aphis gossypii.*

Apart from the conserved *ORs* mentioned above, there are still several species-specific *OR* expansions found in *AgoORs* (Fig. 1). A total of five species-specific clades were found in the phylogenetic tree. The largest *AgoOR* clade contains *AgoOR7, 8, 14, 16, 28, 34* and *36*. Among them, *AgoOR7, 8, 28* and *34* were located tandemly on Scarfold_S000485. Another four sequences in a tandem array, *AgoOR25, 26, 29* and *30*, which are located on Scarfold_S000381, formed another *AgoOR*-specific clade. Furthermore, these sequences in species-specific clades are relatively divergent, as none of them have identity higher than 50% to their most-similar *ApORs*.

**Figure 2:**
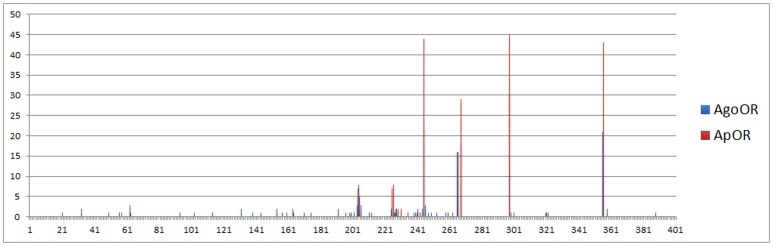
Intron analysis of *A. gossypii* and *A. pisum OR* genes. The intron positions of *A. gossypii* and *A. pisum OR* genes are shown relative to a scale of the average receptor size in amino acids. The x-axis indicates the exon-exon junction positions relative to the amino acid scale of 400aa. The y-axis indicates the number of *ORs* harbouring each exon-exon junction positions.

In addition, we identified 6 intron positions to be conserved among our *AgoOR* sequences, for reason that more than 8 *AgoORs* have introns at these positions. As shown in Fig. 2, the intron position of *AgoORs* and *ApORs* are mapped relative to a scale of the average receptor size in amino acids. 5 amino acid positions were detected as conserved intron positions in both *AgoORs* and *ApORs*. The most conserved intron sites are pos.299, pos.246 and pos.355, for which 45, 44, 43 *ApORs* and 31, 22, 21 *AgoORs* have introns at these sites, respectively. Other conserved positions, pos.267 and 269, were found in both *AgoORs* and *ApORs* of no less than 15 genes. By conjectures from early research, the quantity of OR genes in one species arise through gene duplication, since OR genes are always paired in tandem arrays in the genome and some ancient intron positions are retained in the gene clade [21]. Our intron position analysis conformed to this theory, as well as the tandem *AgoORs* in one scaffold.

### The ionotropic receptors family

The *IRs* in the cotton aphid genome were represented according to their similarity with known insect *IRs*. Bioinformatic analysis led to the identification of 13 candidates *IRs*, in which 11 sequences contain full-length ORFs, the remaining 2 sequences are marked as incomplete because of missing exons. Among these 13 candidate *AgoIRs*, 2 were named as *AgoIR8a* and *AgoIR25a* respectively, for their highly identity to the putative co-operator *DmelIR8a* and *DmelIR25a* (Table 2). 6 of the rest 11 sequences were named as “AgoIR” attached with a number inherited from their *Dmel/Bmor/Slit IR* homologs, as they scored high similarities to their homologs and credible bootstrap values in the phylogenetic test. The remaining five putative *IR* sequences presented neither enough similarity with previously characterized *IRs*, nor reliable bootstrap evidence in phylogenetic analysis. These 5 sequences were named as *AgoIR2, 3, 4, 5* and *6*. The sequences of putative *AgoIR*s are attached in Supplementary Material S2.

**Table 2:** Summary of putative ionotropic receptor genes of A. *gossypii.*

	Sequences				Predicted proteins			Similarity	
gene	status	Length (bp)	aa		Tm domain number	domains		most similar IR	%identity
AgoIR2	3′ exon lost	1125	375		1	S1 S2 P		BmorIR40a	46
AgoIR21a	5′ exon lost	1704	568		5	S1		SlitIR21a	39
AgoIR76b	Complete ORF	1713	571		4	S1 S2 P		DmelIR76b	44
AgoIR3	Complete ORF	1569	523		4	S1 S2 P		DmelIR60a	23
AgoIR4	Complete ORF	1392	464		1	S1 S2 P		BmorIR64a	32
AgoIR68a	Complete ORF	2034	678		4	S1 S2 P		SlitIR68a	42
AgoIR8a	Complete ORF	2595	865		3	S1 S2 P		BmorIR8a	61
AgoIR5	Complete ORF	1992	664		5	NA		BmorIR87a	22
AgoIR75d.1	Complete ORF	2202	734		5	S1 S2 P		SlitIR75d	38
AgoIR75d.3	Complete ORF	1986	662		0	S1 S2 P		DmelIR75d	30
AgoIR6	Complete ORF	2463	821		3	S1		DmelIR76a	27
AgoIR93a	Complete ORF	1860	620		4	S1 S2 P		BmorIR93a	53
AgoIR25a	Complete ORF	2433	811		3	S1 S2 P		BmorIR25a	66

**Figure 3:**
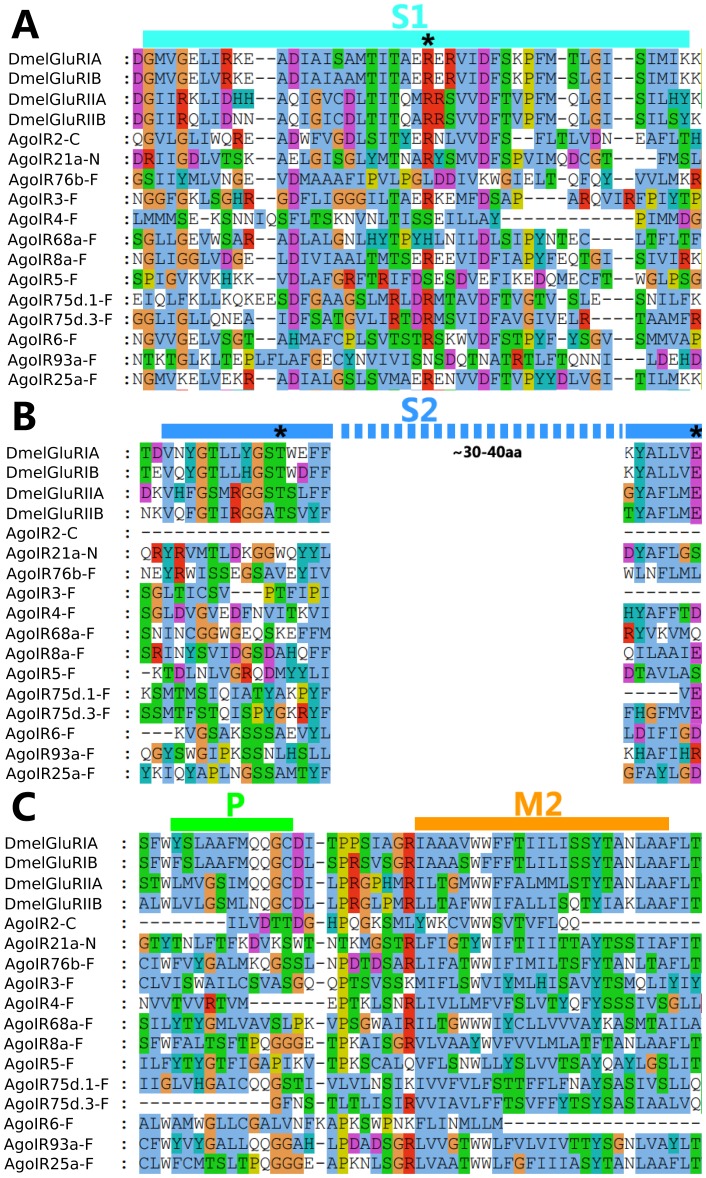
Conserved amino acid positions analysis of Drosophila iGluRs and *A. gossypii* IRs. (A) Amino acid alignments of part of the S1 ligand binding domains. (B) Amino acid alignments of part of the S2 ligand binding domains. (C) Amino acid alignments of part of the pore loop (P) and M2 transmembrane segment of the ion channel domain. The positions of key ligand binding residues in iGluRs are marked with asterisks at the top.

According to the characterization of DmelIRs, the insect IRs contain conserved regions with three transmembrane domains (M1, M2 and M3), a bipartite ligand-binding domain with two lobes (S1 and S2) and one ion channel pore (P) [14]. The most conserved region between IRs and iGluRs spans the ion channel pore (Fig. 3c), suggesting that IRs retain ion-conducting properties. We tested AgoIR sequences in TMHMM, which predicted that the candidate AgoIRs contain transmembrane domains ranging from 0 to 5, and only 3 candidate sequence, AgoIR6, 8, 25 show the typical three transmembrane domains (Table 2). But the protein secondary structure analysis by SMART showed that all AgoIRs except AgoIR21a, 5 and 6 have S1, S2 and PORE domains found. This phenomenon suggests that computational predictions could be inaccurate or that the gene models we annotated are probably incomplete.

Among the conserved domains of iGluRs, the ligand-binding domains containing S1, S2 lobes are considerably more variable. But for the iGluRs family, some conserved amino acid positions were identified to be directly in contact with glutamate or artificial agonists [40]–[42]. Alignment of small regions of the S1 and S2 lobes of AgoIRs revealed that the arginine (R) residue that binds the glutamate α-carboxyl group in the S1 lobe was conserved in eight putative AgoIRs (Fig. 3a); the threonine (T) residue that binds the glutamate γ-carboxyl group in the first half of S2 lob was not retained in any of the 13 AgoIRs (Fig. 3b); a S2 lob end located aspartate (D) or glutamate (E) that interacts with the α-amino group of the glutamate ligand was retained in 6 AgoIRs (Fig. 3b). Thus, no AgoIRs retain the complete set of iGluRs characteristic residues, suggesting that putative AgoIRs bind different ligands.

**Figure 4:**
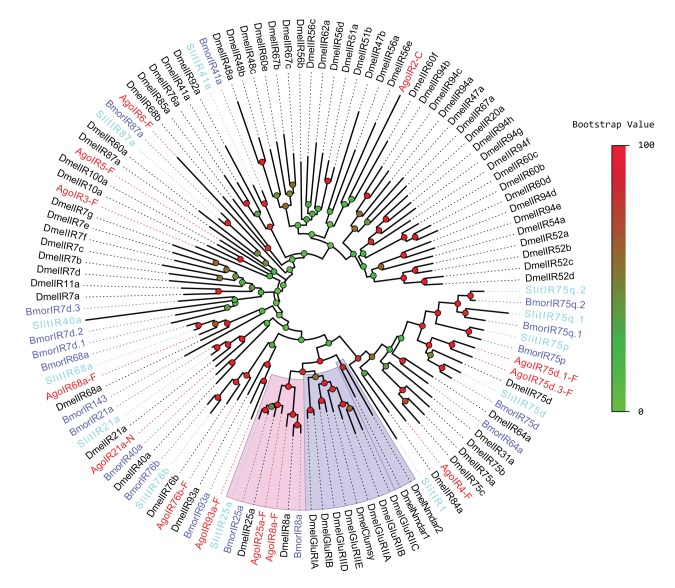
Unrooted tree of putative *AgoIRs*. Ago: *Aphis gossypii*; Dmel: *Drosophila melanogaster*; Bmor: *Bombyx mori*; Slit: *Spodoptera littoralis*. Bootstrap support values presented as percentages were based on 1000 replicates. Accession numbers of sequences used in the analysis were listed in our previous paper (Liu et al., 2012).

To further distinguish putative *IRs* from *iGluRs*, *AgoIRs* were aligned with *IR* orthologues from *D. melanogaster, B. mori, S. littoralis* and some *DmeliGluRs* for phylogenetic analysis. The result revealed a clear segregation between *DmeliGluRs* and insect *IRs* (Fig. 4). The most conserved *IR8a* and *IR25a* clades, which contain *AgoIR8a* and *AgoIR25a*, were confidently clustered together with bootstrap support of more than 80%. Furthermore, amongst the entire *IR* gene family, the *IR8a* and *25a* families contained the closest relatives to the *iGluRs* family, for the reason that they clustered into the big clade of *DmeliGluRs* and *DmelNmdars*. Other putative *AgoIR* sequences we identified did not cluster with *iGluRs* and grouped with other *IRs*.

### Validation by expression profile

**Figure 5:**
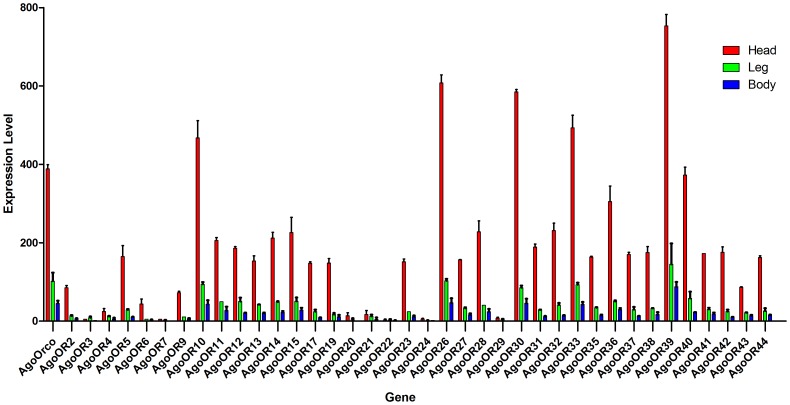
Expression profiles of putative *AgoORs*. The expression was standardized to the expression level of cotton aphid *GAPDH* gene using 2^−ΔΔCt^ method.

**Figure 6:**
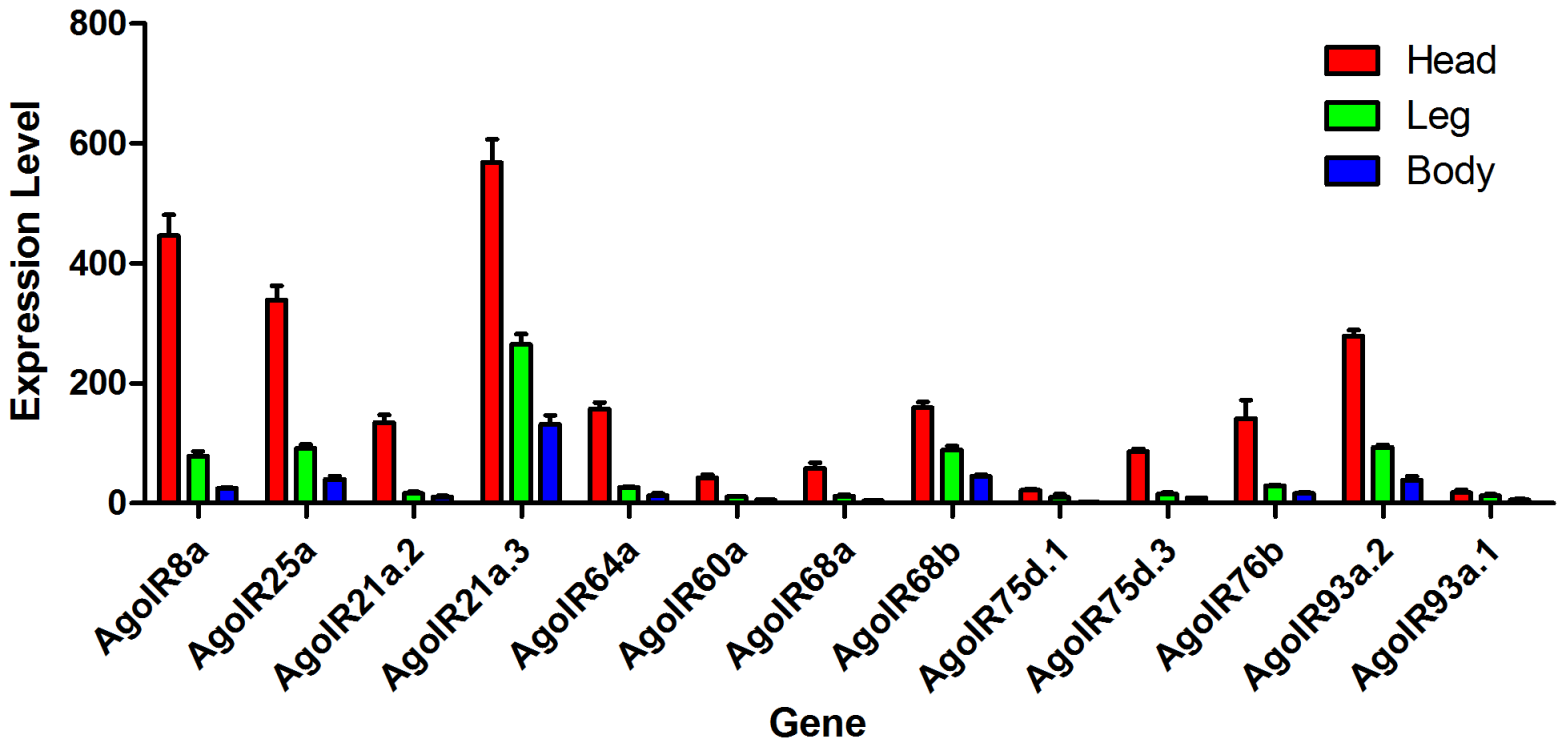
Expression profiles of putative *AgoIRs*. The expression was standardized to the expression level of cotton aphid *GAPDH* gene using 2^−ΔΔCt^ method.

To validate our annotation, we studied the expression of *AgoORs* and *AgoIRs* using qRT-PCR. Of all the 45 putative *ORs*, 38 sequences have expression detected. As shown in Fig. 5, all these putative *ORs* were mainly expressed in the sample “head”, which contains main olfaction organs like antenna and proboscis. The co-receptor gene *AgoOrco* showed a significant higher expression than most of the other *ORs*. This olfaction-organ enriched expression style is consistent with the typical olfaction-gene’s character, proving that the accuracy of our prediction is acceptable. Furthermore, there were 5 *AgoORs* showed a significant higher expression than others, even higher than the co-receptor *AgoOrco*. This phenomenon implied that these high-expression *ORs* may act important roles in the cotton aphid’s chemosensation. For the *AgoIR* dataset, all 13 putative *AgoIRs* were detected with a trend of olfaction-organ enrichment (Fig. 6). Like the *Orco* gene, *IR8a* and *IR25a* genes were thought to act as co-receptors because of their co-expression with other IRs. Our expression profile was consistent with this hypothesis by showing a significant higher expression of *AgoIR8a* and *AgoIR25a* genes. The primers used in our qRT-PCR analysis can be found in Supplementary Material S3.

## Discussion

Previous research on insect *ORs* revealed that odorant receptor genes are highly divergent among different species because *OR* genes undergo stringent Darwinian selection in the process of species formation. Comparing other insects such as *D. melanogaster* and *B. mori*, *OR* genes of cotton aphid indeed display a high degree of divergence. But in comparing *AgoORs* with *ORs* from a closely related species, *Acyrthosiphon pisum*, the result reveals a not-so-divergent relationship. The average identity of *AgoOR-ApOR* pairs is more than 50%, which is significantly greater than another relative species pair, *Ae. aegypti* and *An. Gambiae,* as the majority of *AaORs* share less than 20% identity at the amino acid level with *OR* peptides from *An. Gambiae* [21]. This phenomenon may probably due to the different relative distances between the two aphids and the two mosquitos, but whether other specific characteristics of aphids, such as parthenogernesis or ecological niche, were involved in the evolution procedure of aphid *OR* genes is not known and requires further research.

Compared to the large expansion clades of *ApORs*, the *AgoOR* clades are fewer and less diverse. One obvious reason is that the quantity of *AgoORs* is far less than the *ApORs*. Our phylogenetic tree shows that most of the *AgoORs* are clustered together with *ApORs* and the *AgoOR* specific expansion is rather small. From the common viewpoint that life is a system of maximum energy savings, less genes associated with odorants suggests lower sensitivity to different odorant stimulation. This phenomenon can be easily reconciled with the polyphagous nature of the cotton aphid–fewer odorant receptors make it difficult for the cotton aphid to differentiate between different plants. While for the oligophagous pea aphid, the large family of 73 *ORs* may likely make pea aphid sensitive to the specific odorants of its few host plants.

But what is the relationship between the feeding habit and *OR* quantity? Does the sensitive olfactory system make the pea aphid focus on few plants, or has the longtime process of species formation forced the pea aphid to evolve a complex *OR* family, while this Darwinian selection is relaxed on cotton aphid? We tried to preform selective pressure analysis by PAML using these predicted sequences, and we obtained some interesting results (data not shown). We focused on clades with different components in the phylogenetic trees, including *ApOR*-specific clades, *ApOR-AgoOR*-mix clades and *AgoOR*-specific clades. For the *ApOR*-specific clades, similar results were obtained as Samdja et al [31]–some sequences of the *ApOR*-specific expansion might have evolved under positive selection by one-radio model versus free-radio model. For the *AgoOR-ApOR*-mixed clade, there were still sequences with ω>1 found but the P-Value (after Bonferroni Correction) test stands on the edge of rejection. For the *AgoOR*-specific clade, no sequence was detected with ω>1. Although performing molecular evolution tests on sequences not obtained from strict molecular cloning procedure is unpersuasive, this attempt gave us some ideas about the differences between the species formation of pea aphid and cotton aphid. As the division of *Marcosiphini* and *Aphidini* was thought to happen about 62 million years ago [43], there is reason enough for us to think about whether the olfactory systems of pea aphid and cotton aphid have undergone entirely different types of selection subsequent to parting ways in evolution.

Beyond that, there is still one nonnegligible phenomenon–the pseudogene rate of *AgoORs* is much higher than that in *ApORs*. Sequencing error and mis-assembly could be one reason, and the evaluation requires further research, including cloning and sequencing, transcriptome sequencing of olfactory organs and genome-resequencing. But before these confirmations are done, we should think about the possibility that the pea aphid and cotton aphid have different mechanisms in gene formation, especially in the *OR* family. As is widely agreed, adapting to different hosts is a key factor to the division of aphids. The *gossypii* group of *Aphidini* was formed in the later stage of Oligocene [43] and expanded to over 40 species until now. Conversely, the *Acyrthosiphon* of *Marcosiphini* seems being relatively slow in the step of speciation, as only about 10 *Acyrthosiphon* species were found. However, the current-available taxonomy databases may not represent all aphid species from these families.

The Ionotropic Receptor family, which was recently proposed to detect environmental volatile chemicals in olfactory cilia, is a variant *iGluR* subfamily. Animal iGluRs have been best characterized for their essential roles in synaptic transmission as receptors for the excitatory neurotransmitter glutamate [44], [45]. Three subfamilies with distinct molecular and pharmacological properties were identified in both vertebrates and invertebrates, which are named following their main agonist: a-amino-3-hydroxy-5-methyl-4-isoxazolepropionic acid (AMPA), kainate and N-methyl-D-aspartate (NMDA). IRs share a considerable degree of commonalities with the typical iGluRs: firstly, they are all located to specialized distal membrane domains of neuronal dendrites (cilia and post-synaptic membranes, respectively); secondly, response to binding of extracellular ligands (volatile component and neurotransmitter); thirdly, the multimer form of functional complexes (IR8a/25a co-express with other cell-type specific IRs and the heteromeric assembly of iGluR subunits). It is easy to conjecture that the IR arose from an iGluR with a change in expression localization from an interneuron to a sensilla neuron [15].

Compared to insect *ORs*, the *IR* family is relatively conserved. As the *Orco* is the only homolog gene that discovered extensively among insect species, many *D. melanogaster* antennal *IRs* are conserved in insects, both in sequence and expression pattern, let alone the highly conserved *IR8a* and *IR25a*. There is also evidence in evolution that a *Paleoptera* insect *Rhithrogena semicolorata* bears coeloconic sensilla that contains *IR*-expressing neurons, but no trichoid or basiconic sensilla found [46]. In conclusion, it is conceivable that the *IR* family is a more ancient chemosensory receptor family than the *OR/GR* gene family. And considering that there are fewer *IR* genes, but *IRs* show relatively higher conservation than *ORs*, it is reasonable to think that IRs may probably function to detect molecules being physiologically and behaviorally important to many insect species, and ORs may be primarily dedicated to detection of species-specific odor cues. In our study, orthologues for all 14 putative *IRs* were found in other species, indicating that the AgoIRs may function similarly to *Dmel* antennal IRs, where there is relatively clarity concerning ligands. By far, the known IR ligands include carboxylic acids, ammonia, etc. [47]. Similar compounds have been recorded in GC-EAG analysis or bioassays that can elicit electrophysiological or behavioral response in the aphid [48]–[54]. In all, our 14 putative *AgoIRs* were the first report of *IRs* in the whole family of aphids. This extensively conserved chemosensory receptor family could be a source for new targets of broad-spectrum insect repellents.

## Conclusion

We believe that our approach has thoroughly identified the *OR* and *IR* families in the current version of the *A. gossypii* genome. This enables further investigation of chemosensation in the cotton aphid, in particular explaining the difference in feeding habits between polyphagous and monophagous aphids. The discovery of *ORs* and *IRs* will also assist in the explanation of some classic behaviors like the inter-species alarm behavior and the self-regulation of aphid population, as well as in the discovery of novel volatile compounds, which would give new options for aphid population control by disorientation, mass trapping, or breeding trap crops.

## Supporting Information

Supplementary Material S1 **Sequences of putative** ***AgoOR*** **genes.**
(FASTA)

Supplementary Material S2 **Sequences of putative** ***AgoIR*** **genes.**
(FASTA)

Supplementary Material S3 **Primer sequences used in the quantitative real-time PCR analysis.**
(XLSX)
